# Curcumin enhances cisplatin-induced human laryngeal squamous cancer cell death through activation of TRPM2 channel and mitochondrial oxidative stress

**DOI:** 10.1038/s41598-019-54284-x

**Published:** 2019-11-28

**Authors:** Sinem Gökçe Kütük, Gökçen Gökçe, Mustafa Kütük, Hacer Esra Gürses Cila, Mustafa Nazıroğlu

**Affiliations:** 1 Department of Otorhinolaryngology, Aydın State Hospital, Aydın, Turkey; 2 Department of Histology and Embryology, Faculty of Medicine, Ankara University, Ankara, Turkey; 3 Department of Anesthesiology and Reanimation, Aydın State Hospital, Aydın, Turkey; 4 Department of Molecular Metabolism, Faculty of Medicine, Hacettepe University, Ankara, Turkey; 5 Department of Biophysics, Faculty of Medicine, Suleyman Demirel University, Isparta, Turkey; 6 Drug Discovery Unit, BSN Health, Analysis and Innovation Ltd. Inc. Teknokent, Isparta, Turkey

**Keywords:** Transient receptor potential channels, Apoptosis

## Abstract

In this study, laryngeal tumor cells were killed through the production of excessive reactive oxygen species (ROS) and Ca^2+^ influx by cisplatin (CISP). Nevertheless, a resistance was determined against CISP treatment in the tumor cells. We have investigated the stimulating role of curcumin (CURC) on CISP-induced human laryngeal squamous cancer (Hep2) cell death through TRPM2 channel activation, and its protective role against the adverse effects of CISP in normal kidney (MPK) cells. Hep2 and MPK cells were divided into four groups as control group, CURC group (10μM for 24 hrs), CISP group (25 μM for 24 hrs), and CURC + CISP combination group. CISP-induced decrease of cell viability, cell count, glutathione peroxidase and glutathione level in Hep2 cells were further increased by CURC treatment, but the CISP-induced normal MPK cell death was reduced by the treatment. CISP-induced increase of apoptosis, Ca^2+^ fluorescence intensity, TRPM2 expression and current densities through the increase of lipid peroxidation, intracellular and mitochondrial oxidative stress were stimulated by CURC treatment. In conclusion, CISP-induced increases in mitochondrial ROS and cell death levels in Hep2 cells were further enhanced through the increase of TRPM2 activation with the effect of CURC treatment. CISP-induced drug resistance in Hep2 cells might be reduced by CURC treatment.

## Introduction

The incidence of head and neck tumors is high in malignant carcinomas, and they are the sixth most common type of cancer around the world. About 25% of head and neck tumors are laryngeal carcinomas^1,2^. Hence, the incidence of laryngeal squamous cell carcinoma (LSCC) in the laryngeal tumors is high (98%) among patients, and its incidence has enormously increased despite the use of several environmental protection and drug treatment procedures on the patients^1,2^. For the treatment of laryngeal tumors, chemotherapeutic agents represents an important impact, even though they also have several adverse effects in normal cells^3^. Cisplatin (CISP) is one of the most commonly used drugs among chemotherapeutic agents used for the treatment of LSCC^4^. CISP sensitivity for killing tumor cells is increased by several molecular pathways, including excessive production of reactive oxygen species (ROS)^3,4^ and overload influx of Ca^2+^ ^5,6^. However, resistance to CISP treatment has been observed in patients with laryngeal squamous cancer (Hep2) cell through the imbalance between CISP, Ca^2+^ influx and oxidative stress/antioxidant homeostasis^5,7,8^. Thereby, about 30% of the patients do not respond to initial CISP treatment due to this imbalance^5,7,8^ However, CISP-induced drug resistance was resolved through the increase of ROS production and Ca^2+^ influx in several tumor cells except laryngeal squamous cell carcinoma by the use of some antioxidant supplements such as selenium and alpha lipoic acid^9–11^. Accordingly, we presume that CURC can potentiate the effects of CISP through the inhibition of drug resistance, and the subjects should be examined for Hep2 cells.

Ca^2+^ enables several physiological and pathophysiological functions in body cells^12^. For example, development of normal cells needs Ca^2+^, and excessive Ca^2+^ influx is required for apoptosis in the tumor cells^9,10^. Ca^2+^ concentration is considerably high outside of body cells (1–3 mM) compared to the inside of cells (50–100 nM)^13^. Intracellular free Ca^2+^([Ca^2+^]_i_) concentration is increased in the cytosol through the activation of well-known channels such as voltage gated calcium channels and ligand gated ion channels^13^. In the last decades, new cation channels, namely transient receptor potential (TRP) superfamily, have been discovered^12,13^. The superfamily contains 6 subgroups in mammals, and one subgroup of the TRP superfamily is TRP melastatin (TRPM)^14,15^. TRPM2 is a member of TRPM subgroup, and cation channels are activated by oxidative stress and/or ADPR^16,17^. The increase of intracellular Ca^2+^ concentration is important for killing the tumor cells. In recent studies, some pro-oxidants such as selenium and alpha lipoic acid have enhanced anti-cancer actions of CISP through the activation of TRP channels^9–11^. Accordingly, the similar potentiation action of CURC may be present in the CISP-treated LSCC.

CURC is obtained from turmeric root, and it shows a number of antioxidant, anti-inflammatory and anti-apoptotic actions in normal cells^18^. In recent years, there has been a great interest on the treatment of cancer by CURC since CURC can inhibit cancer tumor growth through inducing tumor apoptosis^18,19^. Accumulating evidence indicates that CURC shows also pro-oxidant and calcium channel activator action in lung cancer cells^20,21^. Due to the significance of enhanced ROS and ROS-activated Ca^2+^ entry (through TRPM2 channel activation) for tumor cell apoptosis, the pro-oxidant action of CURC may enhance CISP efficacy for cancer management.

Till today, there has been no report on the potentiation of CISP-induced apoptosis and oxidative injury in the Hep2 cells by CURC treatment. We have investigated whether CURC synergistically enhanced the anticancer activity of CISP through the activation of TRPM2 channels in the Hep2 cell line. In addition, we have evaluated the possible molecular signaling pathway underlying this effectiveness.

## Results

### Presence of TRPM2 channel in Hep2 cells

**Figure 1:**
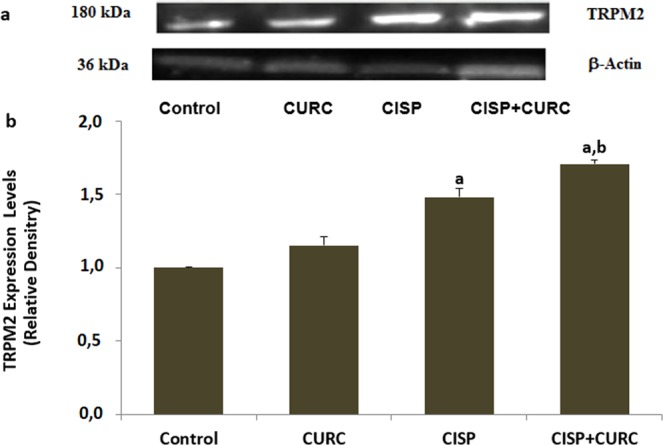
TRPM2 was expressed in the Hep2 cells and its expression level was increased by the cisplatin (CISP) and curcumin (CURC) treatments. (Mean ± SD and n = 3). For detection of TRPM2 channel protein expression level in the frozen Hep2 cells (2 × 10^6^), western blot analysis was used in the four groups. (**a**) representative bands of TRPM2 antibody expression. (**b**) Column figure of TRPM2 channel expression changes. For the equal protein loading, β-actin protein bands were used as control. Imaging, antibody and antigen details of the western blot analyses were given in the material and method section. (^a^p < 0.05 versus control and CURC groups. ^b^p < 0.05 versus CISP group).

It is well known that cell death is induced by several factors. Two of the important ones among these well-known factors are excessive Ca^2+^ entry and oxidative stress^13–15^. Ca^2+^ passes cell membrane through several cation channels. One of these cation channels is the TRP superfamily^13,14^. Some of the subgroup members of TRP superfamily such as TRPA1, TRPM2 and TRPV1 are activated by oxidative stress^6,13–15^. To our knowledge, there has been no report on the presence of TRP channel in Hep2 cell. Therefore, we have scanned the presence of TRPA1, TRPM2 and TRPV1 levels in the Hep2 cell by using Western blot analyses, and found a high expression level of TRPM2 channel in the cells (Fig. 1a). The full length of the Western blots of TRPM2 channel and β-actin are shown in Supplementary Fig. 1. Expression levels of TRPM2 channel in the cells were increased by CISP treatment (p < 0.05). The expression level of TRPM2 was further increased in the CISP + CURC group by CISP plus CURC treatment (p < 0.05).

### CURC treatment enhances cell death effects of CISP in Hep2 cells, but it protects CISP-induced cell death in normal MPK kidney cells

**Figure 2:**
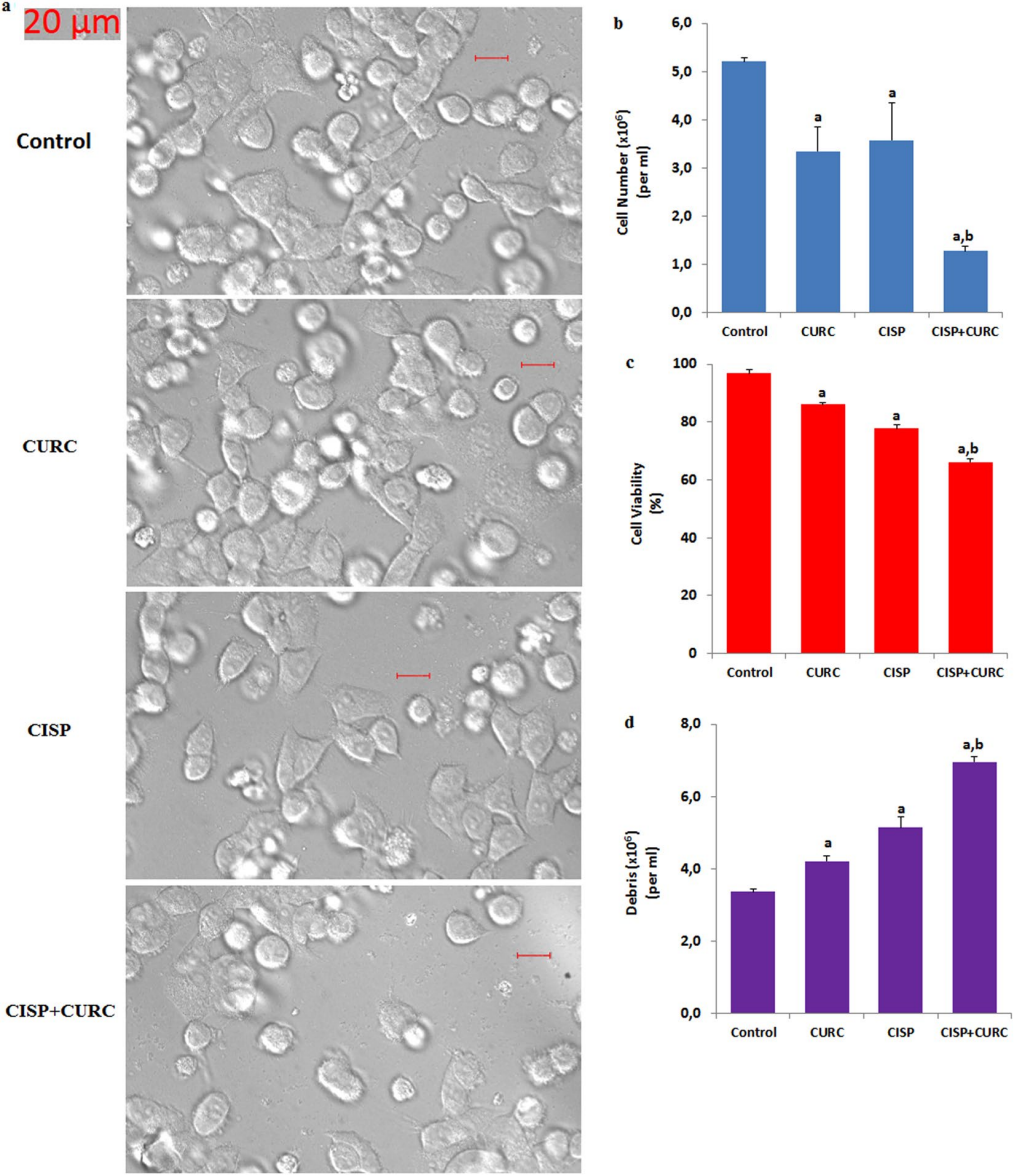
Curcumin (CURC) treatment increased cell death effects of cisplatin (CISP) in the Hep2 cells (mean ± SD and n = 6). Bride field images (**a**) of the cells in the four groups were recorded in Zeiss high-performance sCMOS microscope camera (Axiocam 702 mono) fitted with a 40 x oil objective. Cell number count (**b**), cell viability percentage (**c**), and debris amount (**d**) of the cells were detected by using an electronic counter (Casy TT). (^a^p < 0.05 versus control. ^b^p < 0.05 versus CURC and CISP groups).

In the Hep2 results, cell death debris levels were markedly enhanced by CISP although cell viability level and cell count numbers were decreased by CISP treatment (p < 0.05) (Fig. 2a–c). In addition, cell debris level was further enhanced in CISP + CURC group by CURC treatment, although cell viability level and cell count numbers were further decreased in Hep2 cells by CURC treatment (p < 0.05) (Fig. 2d).

**Figure 3:**
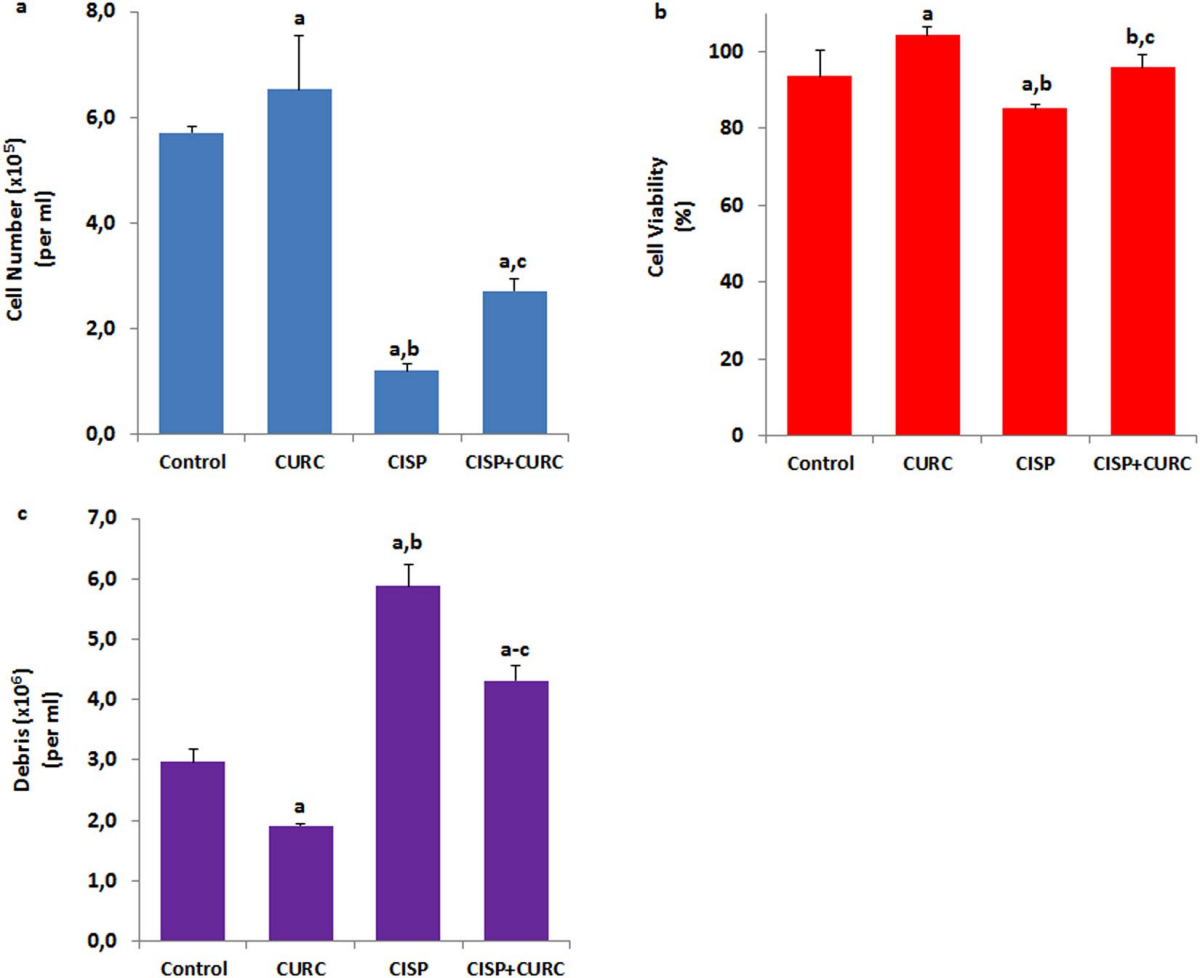
Curcumin (CURC) treatment protected normal kidney (MPK) cells against cisplatin (CISP)-induced cell death (mean ± SD and n = 6). Cell number count (**a**), cell viability percentage (**b**), and debris amount (**c**) of the cells were detected by using an electronic counter (Casy TT). (^a^p < 0.05 versus control. ^b^p < 0.05 versus CURC group. ^c^p < 0.05 versus CISP group).

In addition to cancer (Hep2) cells, we have investigated protective or toxic effects of CURC against CISP treatment in normal kidney (MPK) cells. Contrary to Hep2 cell viability results, CISP treatment has decreased the levels of cell number count and cell viability rate (p < 0.05) compared to control, although the CISP + CURC treatment increased this number and rate in normal kidney cells (Fig. 3a,b). In parallel with the cell number and viability results, cell death debris rate was increased in the CISP group for the kidney cells compared to control group, although its rate was reduced in CISP + CURC groups by the effect of CURC treatment (Fig. 3c). More importantly, we have found that CURC increased the level of cell viability and cell number in normal kidney cells, but it decreased the level of cell viability and cell number in Hep2 cells. Our data has suggested that CISP-induced cell death effect could be (cell-specifically) further enhanced by CURC treatment.

### CURC increases TRPM2 channel currents in Hep2 cells

After observing TRPM2 channel in Hep2 cell, we have investigated the role of excessive Ca^2+^ entry through TRPM2 in CISP- and CURC-treated Hep2 cells by using patch-clamp analyses.

**Figure 4:**
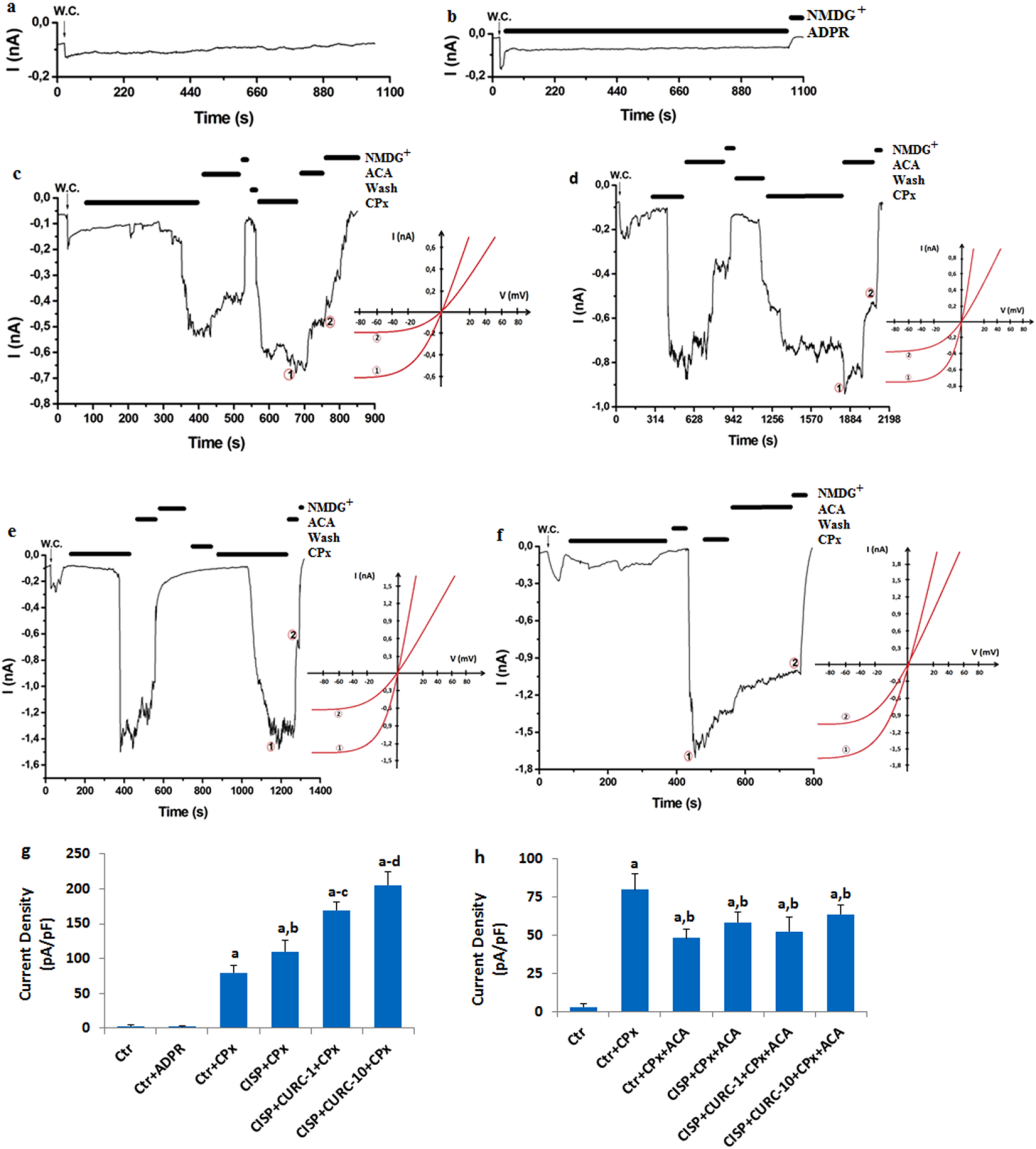
Curcumin (CURC) treatment increased cisplatin (CISP)-induced TRPM2 current densities in the Hep2 cells (mean ± SD and n = 3). (**a**) Control records. (**b**) Control + ADPR. (**c**) Control + CPx (without CISP and CURC). (**d**) CISP + CPx. The cells in the group were incubated with CISP (25 μM) for 24 hours. (**e**) CISP + CURC-1-CPx. The cells in the group were treated with CURC (1 μM) for 24 hours and then they were incubated with CISP (25 μM) for 24 hours. (**f**) CISP + CURC-10-CPx. The cells in the group were treated with CURC (10 μM) for 24 hours and then they were incubated with CISP (25 μM) for 24 hours. All groups (**c**–**f**) except control and control + ADPR were stimulated with 10 mM cumene hyroperoxide (CPx). (**g**) current densities of CPx groups. (**h**) current densities of ACA groups. ^a^p < 0.05 versus Ctr. ^b^p < 0.05 versus Ctr + CPx. ^c^p < 0.05 versus CISP + CPx. ^d^p < 0.05 versus CISP + CURC + CPx).

TRPM2 channel is activated by intracellular ADP-ribose (ADPR) and extracellular oxidative stress^16,17^. In all cell patch-clamp analyses, TRPM2 channel in Hep2 cells has been stimulated by intracellular ADPR (1 mM) and extracellular cumene hydroperoxide (CPx and 10 mM), although they were blocked by TRPM2 channel blocker (ACA and 25 μM). First, we checked all buffers and patch-clamp conditions by taking a control record without any stimulation through ADPR and CPx. We have observed no current in the control record (Fig. 4a). Then, we tested intracellular ADPR stimulation, and we could not find TRPM2 activation in the Hep2 cells due to the activating effect of intracellular ADPR (Fig. 4b). Afterwards, we have tested the effects of extracellular CPx on the TRPM2 channel activation in Hep2 cells. The cells were activated in Hep2 cells by extracellular CPx stimulation without CISP and CURC treatment (Fig. 4c). In addition, we have observed enhanced TRPM2 current densities in the CISP group by extracellular CPx treatment (Fig. 4d), and their current densities were higher in the CISP + CPx group than in the control + CPx group (p < 0.05) (Fig. 4g). Current densities were further enhanced in CISP + CURC groups by CPx stimulation (Fig. 4e,g) and CURC (10 μM) treatments (Fig. 4f,g) (p < 0.05). CISP-induced TRPM2 channel activity was further enhanced in Hep2 cells by CURC treatment (p < 0.05) (Fig. 4g). However, TRPM2 current densities were blocked by TRPM2 channel blocker (ACA), and their current densities were markedly (p < 0.05) lower in CPx + ACA groups compared to CPx group (Fig. 4h).

### CURC enhanced CISP-induced increase of Ca^2+^ florescence intensity through TRPM2 activation in Hep2 cells

**Figure 5:**
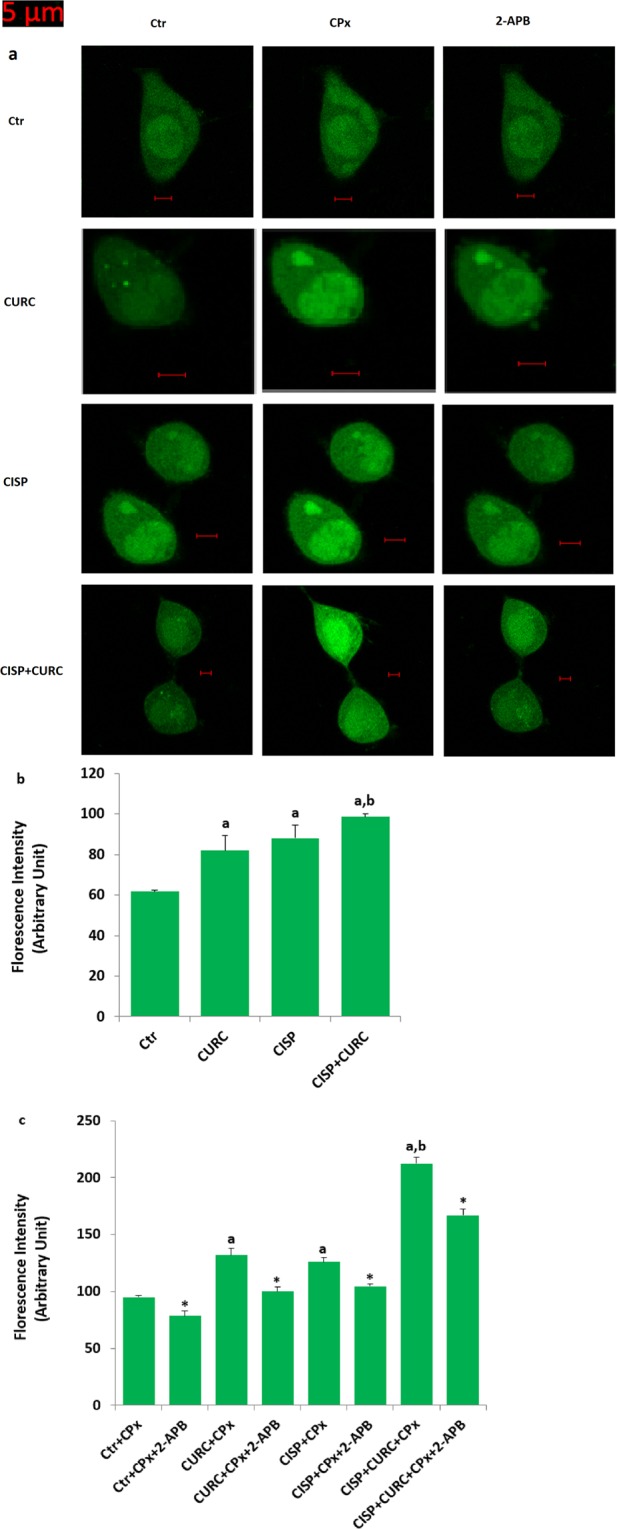
Curcumin (CURC) treatment increased cisplatin (CISP)-induced Ca^2+^ influx through activation of TRPM2 channels in the Hep2 cells (mean ± SD and n = 20–25). The cells in the group CURC and CISP groups were incubated with CURC (10 μM) and CISP (25 μM) for 24 hours, respectively. In the CURC + CISP group, the cells were incubated with CURC (10 μM) for 24 hours and then they were further incubated with CISP (25 μM) for 24 hours. Cells in the four groups were stimulated by CPx (1 mM) and they were blocked by 2-APB (100 μM). The cells were stained with Fluo-3 calcium dye and mean ± SD of fluorescence in 12 mm^2^ of the cells as arbitrary unit are presented (**a**). The cells were extracellularly stimulated by CPx (1 mM for 10 min), but they were inhibited by 2-APB (100 μM for 10 min) (**a**). The samples were analyzed by the laser confocal microscopy fitted with a 40 x oil objective. Changes of the Ca^2+^ fluorescence intensity were shown by columns (**b** and **c**). (^a^p* < *0.05 versus control (Ctr). ^b^p < 0.05 versus CURC, CISP, CURC + CPx and CISP + CPx groups. *p < 0.05 is indicating effects of 2-APB).

In addition to patch-clamp electrophysiology analyses, we wanted to further clarify the involvement of TRPM2 channel on Ca^2+^ influx in Hep2 cells by using laser confocal microscopy imaging analyses. Similar to patch-clamp results, intracellular Ca^2+^ fluorescence intensity through TRPM2 channel activation (CPx stimulation) was higher in CISP group compared to control group (p* < *0.05) (Fig. 5a,b). CISP and CURC combination treatment further enhanced the increased level of Ca^2+^ fluorescence intensity (p* < *0.05) (Fig. 5a,b). However, CPx-induced increases of intracellular Ca^2+^ fluorescence intensity were decreased in control, CURC, CISP and CURC + CISP groups by the TRPM2 antagonist (2-aminoethyl diphenylborinate, 2-APB) pretreatment (Fig. 5c). These imaging results confirmed the effect of CURC on excessive Ca^2+^ influx that is mediated by TRPM2 channel in the cells.

### Curcumin (CURC) stimulated CISP-induced increase of intracellular and mitochondrial ROS production in Hep2 cells

Mitochondria is a main source of ROS production. In addition, the accumulation of free Ca^2+^ in the mitochondria induces increases in electron transport system, resulting in mitochondrial membrane depolarization **(**MMD) production^22^. In turn, the increase of MMD induces increases in mitochondrial and intracellular ROS productions^9,10^. For this reason, MMD is an important parameter of mitochondrial function and ROS production, and it has been used as an indicator of ROS production in normal and cancer cells^23,24^.

**Figure 6:**
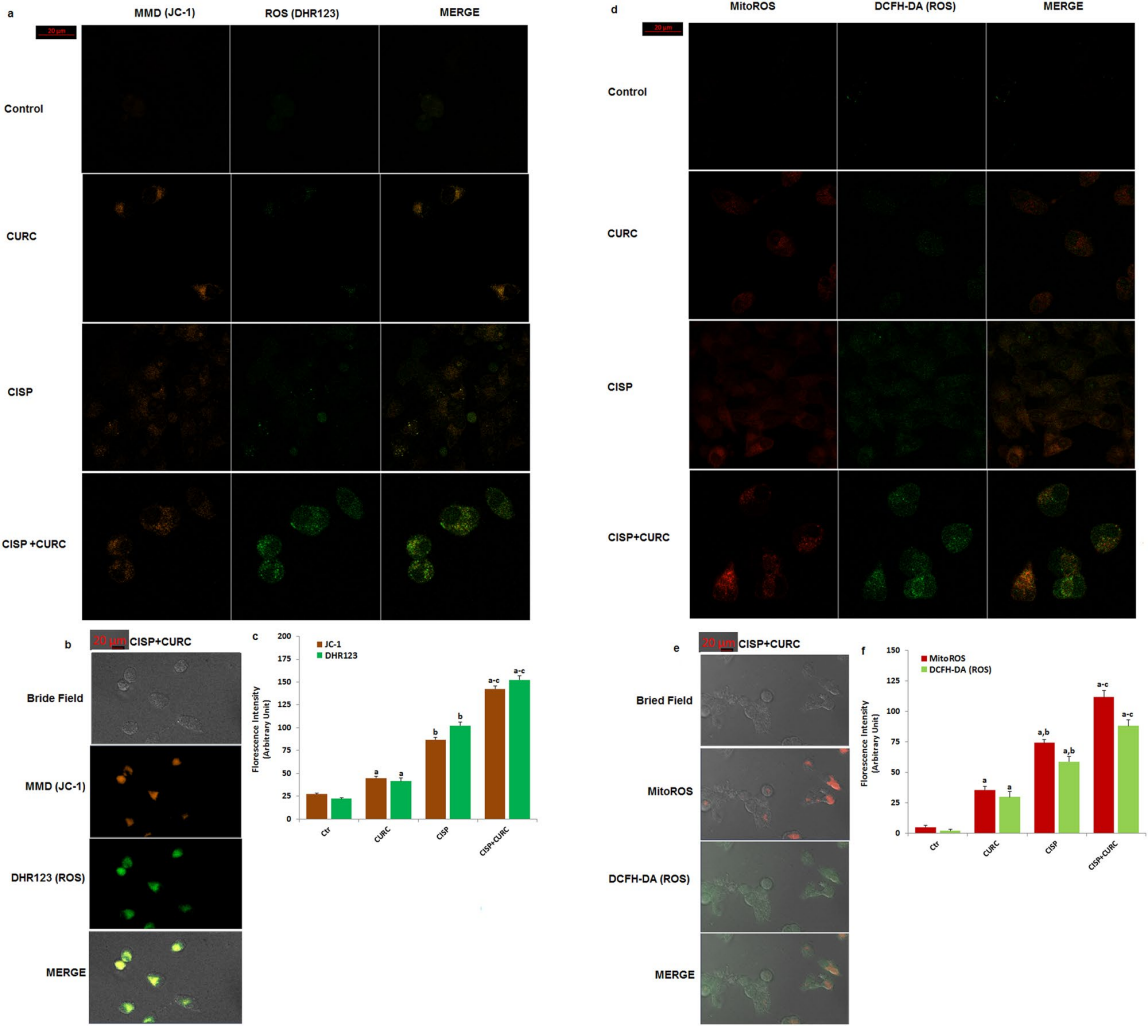
Cisplatin (CISP)-induced increases of mitochondrial membrane depolarization (MMD), intracellular ROS and mitochondrial ROS (MitoROS) in the Hep2 cells were further increased by the curcumin (CURC) treatment. (mean ± SD and n = 20–25). The cells in the group CURC and CISP groups were incubated with CURC (10 μM) and CISP (25 μM) for 24 hours, respectively. In the CURC + CISP group, the cells were incubated with CURC (10 μM) for 24 hours and then they were incubated with CISP (25 μM) for 24 hours. The cells were stained with 1 μM MMD dye (JC-1) (**a**–**c**), cytosolic ROS [DHR123 (**a**–**c**) and DCFH-DA (**d**–**f**)] and mitochondrial (MitoTracker Red CM-H2ros ROS staining (MitoROS) (**d**–**f**) production indicators Fluorescence intensities of the four dyes in 12 mm^2^ of the cells are presented as arbitrary units by columns (**b** and **d**). The samples in the **a** and **d** were analyzed by the laser confocal microscopy fitted with a 40 x oil objective. However, the 3D bride field images of cells (**b** and **e**) in the CISP + CURC group were taken with a Zeiss high-performance sCMOS microscope camera (Axiocam 702 mono) with a 40 x oil objective. (^a^p* < *0.05 versus control (Ctr) group. ^b^p < 0.05 versus CURC group. ^c^p < 0.05 CISP group).

After observing the increase in intracellular Ca^2+^ concentration through TRPM2 channel activation, we suspected whether intracellular and mitochondrial ROS production increased through the increase of MMD. Therefore, we have aimed to analyze the changes in MMD and ROS in the cells. In several cells, JC-1 has been used in laser confocal microscope (LSM 800) for analyzing MMD^9,10,22,25^. Intracellular (DHR123 and DCFH-DA) and mitochondrial (MitoROS) ROS productions were also assayed in the cells by using appropriate dyes^23,24^. The MMD (Fig. 6a,b), DHR123 (Fig. 6a,b), DCFH-DA (Fig. 6c,d), MitoROS (Fig. 6c,d) levels were increased in the cells with the effect of CISP treatment (p < 0.05). In addition, CURC co-treatment further increased the effect of CISP through stimulation of mitochondrial ROS in the cells (p < 0.05) (Fig. 6e,f). These imaging results further supported the effect of CURC on TRPM2 channel activation-induced mitochondrial oxidative stress activity in the Hep2 cells.

### CURC treatment stimulates CISP-induced cancer cell death in Hep2 cells

**Figure 7:**
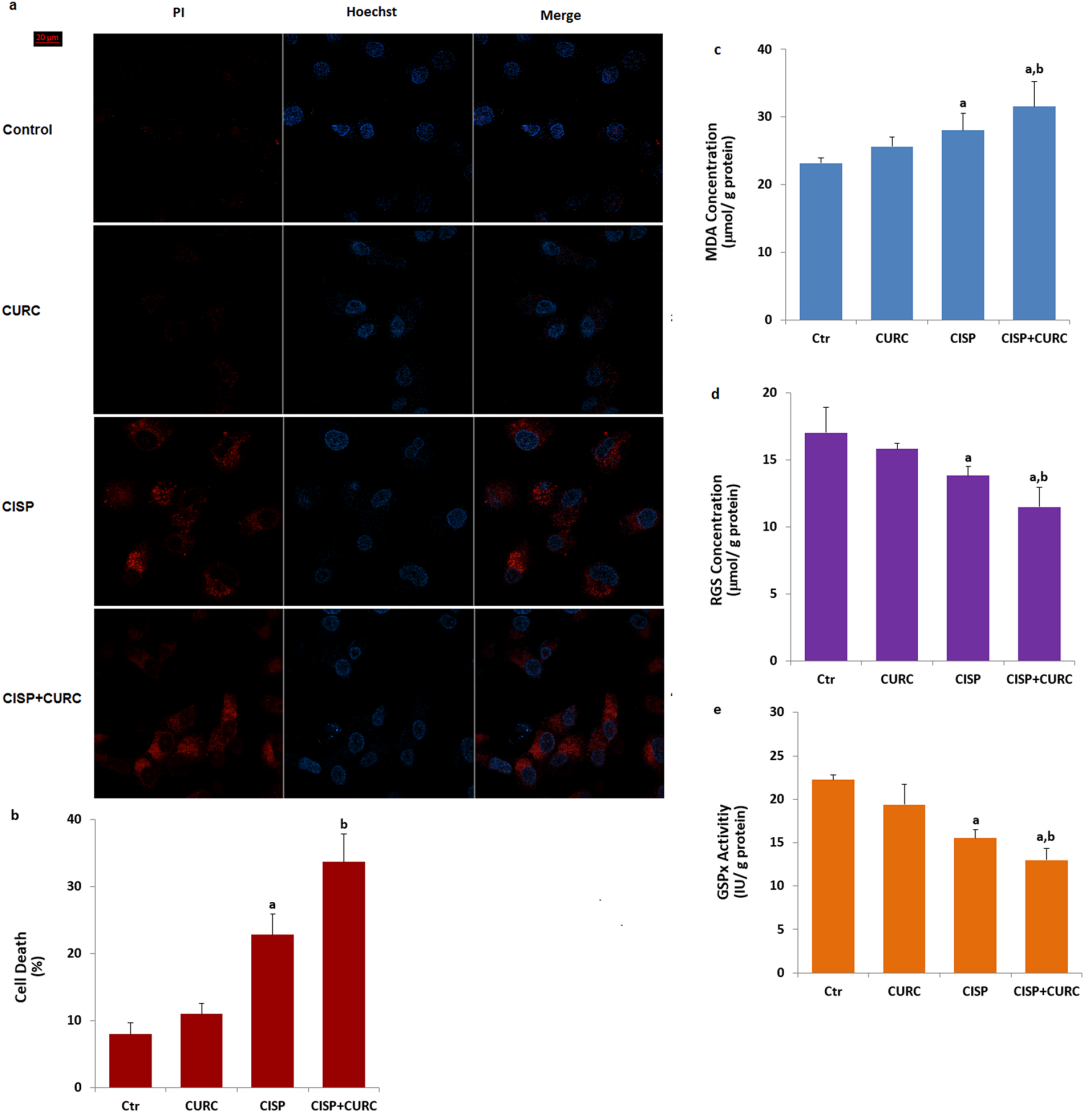
Curcumin (CURC) and cisplatin (CISP) increase Hep2 cell death and lipid peroxidation (MDA) through down regulation of reduced glutathione (RGS) level and glutathione peroxidase (GSPx) activity. (**a**,**b**) are representative images showing dead (propidium iodide, PI) and live (Hoechst 33342) staining of Hep2 cells under control conditions or after exposure to CURC (10 μM) and CISP (25 µM) and their combinations. Each panel consists of PI (red) and Hoechst (blue)-staining images showing dead and live cells and merged Hoechst (blue)/PI-staining image showing all and dead cells. The scale bar is 20 μm. (**b**) Summary of the mean percentage of PI and Hoechst-positive cells under the indicated conditions from 6 independent experiments, with each experiment examining 20–25 cells for each condition. The red bars represent the percentage of Hep2 cell death. We also assayed MDA, RGS concentration and GSPx activity in the four groups by using a spectrophotometer (**c**–**e**). CISP-induced increase of MDA levels was further increased by down regulation of RGS level and GSPx activity. (^a^p < 0.05 versus control (Ctr) and CURC groups. ^b^p < 0.05 versus CISP group).

It is well known that increased mitochondrial ROS activation due to the activation of TRPM2 channels in cancer cells results in cell death and apoptosis^6,26^. After observing the increase in mitochondrial ROS and TRPM2 channel, we suspected whether cell death was increased in the cancer cells. Propidium iodide (PI) is an indicator of dead cells, while Hoechst dye is indicator of live cells (Fig. 7a). The percentage of dead cells was increased by CISP treatment (p < 0.05) (Fig. 7b). The increase of cell death was further increased by CURC treatment and its percentage in CISP + CURC group were markedly (p < 0.05) higher than CISP group (Fig. 7b). For this reason, we have observed the effect on cell death by CURC in Hep2 cells.

### Curcumin (CURC) treatment supports glutathione redox system for scavenging cisplatin (CISP)-induced excessive ROS production in Hep2 cells

ROS are scavenged by antioxidants^12,15^. Members of thiol redox system such as glutathione and GSPx play the main role for the scavenging of ROS in several cells^27,28^. CURC supports RGS concentration and GSPx activity in normal cells^29,30^. Thiol groups also play a main role in the activation of TRPM2 channel and RGS depletion in neurons induced by the excessive activation of TRPM2 channel through the increase of oxidative stress^31,32^. After observing an increase in ROS levels, we have considered that decreased glutathione concentration and GSPx activity may induce the activation of TRPM2 channel in the Hep2 cells. The lipid peroxidation levels as MDA (Fig. 7c) were increased by the CISP and CURC treatments in the Hep2 cells. In addition, CISP-induced decrease of RGS concentration (Fig. 7d) and GSPx (Fig. 7e) activity in the Hep2 cells was further decreased by CISP + CURC treatment (p < 0.05). These RGS and GSPx results confirmed the glutathione depletion effect through excessive oxidative stress, mediated by TRPM2 channel activation in the Hep2 cells.

## Discussion

**Figure 8:**
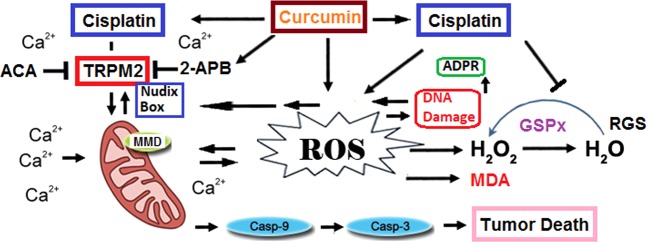
Summary of pathways involved in cisplatin and curcumin-induced TRPM2 channel activation and tumor cell death through excessive reactive oxygen species (ROS). Cisplatin can lead in Hep2. Nudix box domain of TRPM2 channel is sensitive to ROS. ROS-induced TRPM2 activation stimulates excessive Ca^2+^ influx. ROS are produced in the mitochondria through the influx of excessive Ca^2+^. The main mechanism in the cell death effect of curcumin is mediated by stimulation of ROS-mediated caspase -3 and -9 activations. In response, cisplatin and curcumin in the Hep2 cells initiates a pro-oxidant response that stimulates the ROS and lipid peroxidation (MDA) into less harmful products by inhibiting reduced glutathione (RGS) and glutathione peroxidase (GSPx).

In this study, CISP-induced decreases in cell viability and cell count values of Hep2 cells were enhanced by CISP and CURC treatments, but the values were inhibited in normal kidney (MPK) cells with the effect of these treatments. CISP induced increases in TRPM2 channel current density, expression level, Ca^2+^ fluorescence intensity, lipid peroxidation, MMD, caspase activity, and cytosolic and mitochondrial ROS production, but its treatment induced decreases in RGS level and GSPx activity in Hep2 cells. Therefore, CISP applications are characterized in the Hep2 cell by increases in TRPM2 channel and caspase activation through excessive ROS production, but also decreases in tumor cell viability, RGS and GSPx. However, TRPM2 activation, and intracellular and mitochondrial ROS production were further enhanced by CURC treatment, while cell viability and GSPx were further decreased by the combination (CISP + CURC) treatment. The current results are the first to compare the effect of CURC on CISP-induced TRPM2 channel activation and cell death through excessive ROS production in Hep2 tumor cells (Fig. 8).

We first investigated the changes in cell viability, cell number and cell debris levels in CISP- and CURC-treated Hep2 tumor and MPK kidney normal cells. We have observed cell-specific anti-tumor action of CURC in the cells. Tumor cells are killed by several molecular mechanisms, including excessive Ca^2+^ influx and ROS production^4–6^. Therefore, we have decided to investigate the changes in intracellular Ca^2+^ concentration in Hep2 cells. Best candidate for investigating the changes in intracellular Ca^2+^ concentration was TRPM2 channel due to two reasons; (1) its expression in Hep2 cells (Figs. 1 and 2) it is activated by ADPR and oxidative stress^16,17^. In the patch-clamp experiments and laser confocal microscope analyses, we have observed increased levels of TRPM2 current density and Ca^2+^ fluorescence intensity through oxidative stress (CPx) stimulation in Hep2 cells, but their density and intensity were decreased by TRPM2 channel blockers (ACA and 2-APB) in Hep2 cells. In recent studies, the protective role of CURC through the inhibition of TRPM2 channel were reported against excessive Ca^2+^ entry into cytosol of different normal cells^29,30,33–36^. However, accumulating evidence indicates that CURC has also pro-oxidant and calcium channel activating actions^20,21,37,38^. Due to the importance of enhanced ROS and ROS-activated TRPM2 channel activation on tumor cell death, we have investigated the effect of CURC on CISP-induced TRPM2 current density, TRPM2 expression level and Ca^2+^ fluorescence intensity values through oxidative stress stimulation in the Hep2 cells. We have observed that CISP-induced TRPM2 current density, expression level and Ca^2+^ fluorescence intensity in Hep2 cells were further enhanced by CURC treatment. Therefore, our results have confirmed the reports on pro-oxidant and calcium channel activator action of CURC in tumor cells^20,21,37^.

It is well-known that increases in intracellular free Ca^2+^ concentration induces increased MMD in normal and tumor cells^38,39^. In addition, CISP and CURC-induced TRPM2 channel might be activated in Hep2 cell by increases in intracellular and mitochondrial ROS production. Hence, we have investigated the involvement of CISP and CURC on lipid peroxidation and caspase activation due to increases in intracellular and mitochondrial ROS productions in Hep2 cell. MMD (JC-1), lipid peroxidation, intracellular ROS (DHR123 and DCFH-DA) and mitochondrial ROS (MitoROS) levels were increased in Hep2 cells with CISP treatment. In addition, the values were further enhanced by CURC + CISP treatment. Increases were determined for MMD results in two molecular pathways; (1) increase in intracellular ROS production and (2) increase in cell death^9,10^. Therefore, there is a direct relationship between the increase of MMD-induced ROS and the increase in cell death^38^. Similarly, previous reports showed that apoptosis level was increased in tumor lines by the upstream of CISP-induced MMD^9,10,38^. When CISP induces an overload of mitochondrial ROS generation, pro-apoptotic proteins in Hep2 cells are activated to induce MMD, releasing the caspases -3 and -9 activations^39,40^. For this reason, cell death through the stimulation of ROS production and MMD was further increased in the tumor cells with CURC treatment^41^.

The thiol redox system has a major role in scavenging ROS, two main members of the thiol redox system being RGS and GSPx. Decreased levels of RGS and activity of GSPx have been reported in laryngeal cancer patients, and their values were further decreased after CISP treatment^42^. In addition, polymorphisms were reported in RGS and GSPx in head and neck squamous cell carcinoma of patients^43^. Depletion of RGS is important for the activation of TRPM2 channel^31,32^. For scavenging ROS, depletion of RGS and GSPx values might have occurred in CISP- and CURC-treated Hep2 cells. Hence, we have analyzed the changes in RGS and GSPx values in Hep2 cells. The CISP-induced decrease of RGS level and GSPx activity were further stimulated in Hep2 cells with CURC treatment. Similarly, decreased RGS level was reported in Hep2 cells with CISP treatment due to increases in apoptosis level and ROS production.

In summary, the results presented in this study indicate that CURC increases the chemotherapeutic action of CISP against Hep2 cells by inducing oxidative stress, and suppressing cell viability and number. More importantly, these alterations were arranged by TRPM2 channel-activation-induced excessive Ca^2+^ influx and mitochondrial oxidative stress pathways. Overall, our results indicate that combination treatment of CURC and CISP could provide an improved strategy for CISP chemotherapy and may be related with the TRPM2-associated increases in the excessive oxidative stress and Ca^2+^ influx.

## Materials and Methods

### Reagents

Curcumin (CURC), cisplatin (CISP), dimethyl sulfoxide (DMSO), cumene hydroperoxide (CPx), ADPR, Dihydro-rhodamine 123 (DHR 123), 2′,7′-dichlorodihydrofluorescein diacetate (DCFH–DA), malondialdehyde, reduced glutathione (RGS), sodium pyruvate and L-glutamine were purchased from Sigma Aldrich (St. Louis, MO, USA). Two TRPM2 blockers, N-(p-Amylcinnamoyl) anthranilic Acid (ACA) and 2-aminoethyl diphenylborinate (2-APB) were purchased from Santa Cruz (Istanbul, Turkey). Fluo-3, Hoechst 33342, propidium iodide and MitoTracker Red CM-H2ros staining (MitoROS) were purchased from Life Technologies (Istanbul, Turkey). Dulbecco’s modified Eagle’s medium (DMEM) and flasks were purchased from Thermo Fischer Sci. Inc. (Istanbul, Turkey), and fetal bovine serum was purchased from Cegrogen Biotech (Stadtallendorf, Germany). Glass bottom dishes were purchased from Mattek Corporation Inc. (Ashland, MA, USA). Antibodies, antigens and β-actin were purchased from Santa Cruz Inc. (Istanbul, Turkey). Nitrocellulose membrane was purchased from BioRad Laboratories Inc. (Istanbul, Turkey).

### Cell culture

We have used Hep2 cell line in this study, which was originally produced in Şap Institute, (Ankara, Turkey). The cells were cultured in a medium consisting of DMEM with low glucose (1 g/l). Fetal bovine serum, antibiotics, and the appropriate supplements such as sodium pyruvate and L-glutamine.

In this study, we have also used murine kidney cortical collecting ducts mpkCCD_c14_ (MPK) cells as normal cells (gift from Anatomy, Department of Medicine, Fribourg University, Basel, Switzerland), due to the fact that we have recently determined high TRPM2 expression levels in the cells^34^. The MPK cells were cultured in defining the medium as described previously^44^. In brief, the growth medium was composed of DMEM (50%) and Ham’s F12 (50%).

Both cells were grown in a 5% CO_2_/ 95% air atmosphere at 37 °C. Cells were either treated with CURC and CISP. Used concentration level was indicated in the figure legends. In some experiments, specific TRPM2 blockers (ACA and 2-APB) and stimulator (CPx and 1–10 mM) were applied in addition to the culture media used^45^.

### Groups

In the western blot analyses of this study, we have observed high expression levels of TRPM2 channel in Hep2 (Fig. 1) and kidney MPK cells^34^. For this reason, these cells were used for the investigation of the channels in this study. The cells were mainly divided into four groups as described below;

#### Control group

The cells were not incubated with CURC and CISP, but were kept in a flask containing the same cell culture medium and conditions for 48 hours.

#### CURC group

The cells in this group were kept for 24 hours in the same cell culture condition without applying treatment, and then they were pre-incubated with CURC (10 μM) for 24 hours as described in a previous studies^25,34,46^.

#### CISP

The cells in this group were kept for 24 hours in the same cell culture condition without applying treatment, and then they were incubated with CISP (25 μM) for 24 hours^9,26^.

#### CURC + CISP group

The cells in this group were kept with CURC (10 μM) in the cell culture medium for 24 hours, and then they were treated with CURC (25 μM) further for a period of 24 hours.

Stock CURC solution (1 mM), ACA and 2-APB (1 mM) was dissolved in DMSO and they were diluted to the appropriate concentrations (25 μM) and (0.1 mM) in DMEM, respectively.

#### Assay of cell viability, cell count and debris

The cell viability, cell count and debris levels were assayed by using an automatic cell counter (Casy Modell TT, Roche, Germany) as described in a previous study^25^. The main principle of the device is the fact that cells displace a quantity of electrolytes corresponding to their volume during their passage through the measuring pore. The results of cell viability were expressed as % change, although debris amount and cell count was expressed as x10^6^/per ml.

### Electrophysiology

In the patch-clamp analyses, the cells were seeded in 6–8 flasks at a density of 1 × 10^6^ cells per flask (filter cap, sterile, 260 ml, 80 cm²). In Hep2 cells, whole-cell voltage clamp recording was taken at room temperature by using EPC10 patch-clamp set (HEKA, Lamprecht, Germany). Details of the extracellular and intracellular buffers were provided in the previous studies^9,10^. It has been reported that TRPM2 channel is activated in the presence of high intracellular Ca^2+^ ^47^. Therefore, intracellular Ca^2+^ concentration in the intracellular buffer was kept as 1 μM instead of 0.1 μM. The cells were held at −60 mV potential for voltage clamping. Ramps (current-voltage relationships) were obtained from −200 to + 200 mV applied over 300 milliseconds. The maximal current amplitudes (pA) in Hep2 cell were divided by the cell capacitance (pF), which is a measure of the cell surface. Values of current density were expressed as pA/pF in the experiment. ADPR is an activator of TRPM2 channel^17^, although ACA is an inhibitor of TRPM2^27,48^. Hep2 cells were stimulated by intracellular ADPR (1 mM) and CPx (10 mM), but they were inhibited by ACA (25 μM).

### Measurement of intracellular Ca^2+^ fluorescence intensity through TRPM2 activation

It is well known that labeled Ca^2+^ indicators exhibit increases in fluorescence upon binding Ca^2+^. One of the indicators of that is Fluo-3, AM and it has been used to image the spatial dynamics of Ca^2+^ signaling. Changes in intracellular Ca^2+^ fluorescence concentration in Hep2 cell were monitored by fluorescence changes of Fluo-3 (Calbiochem, Darmstadt, Germany) as described in a previous study^25^. First, the cells were seeded in 35 mm glass bottom dishes. Then, after incubating the cells with Fluo-3 (1 μM for one hour in the dark), the dye was excited by a 488 nm argon laser from the confocal microscope (LSM 800, Zeiss, Ankara, Turkey)^25^. In some of the experiments, the cells were treated with TRPM2 antagonist (2-APB and 100 μM) to inhibit Ca^2+^ entry before CPx stimulation (1 mM)^49^. The cells were analyzed at 515 nm by laser confocal microscopy fitted with a 40x oil objective. The results of fluorescence intensity in 10 μm^2^ of cytosol were measured in the laser confocal microscope by using ZEN program. The mean fluorescence intensity was expressed with an arbitrary unit per cell.

### Imaging of ROS generation and MMD (JC-1) in Hep2 cells by laser confocal microscope analyses

For the imaging of JC-1 in the cells, the cells were incubated with JC-1 (5 μl) fluorescent dye for 15 minutes at 37 °C in the dark. The samples were then analyzed by laser confocal microscopy (LSM 800). JC-1 dyes in the cells were excited with a diode laser at 488 nm, an Argon laser at 488 nm^22^. Fluorescence intensity (arbitrary unit) of each cell as was recorded by using ZEN program and analyzed using ImageJ/Imaris software. The results of JC-1 were expressed as the mean fluorescence intensity in arbitrary unit/cell.

Mitochondrial ROS generation in the laser confocal microspore analyses (LSM 800) was assayed by using MitoTracker Red CM-H2Xros florescent dye according to manufacturer’s instructions. Intracellular ROS production was monitored by two fluorescent indicator dyes (DHR123 and DCFH-DA). After being exposed to these treatments, the cells were incubated in a culture medium containing 100 nM MitoTracker Red CM-H2Xros for 30 minutes, and 1 μM DCFH-DA or DHR123 for 20 minutes at 37 °C in dark^23,24^. The cells were washed with and maintained in 1xPBS before their imaging. DHR123 and DCFH-DA dyes were excited with a diode laser at 488 nm. ZEN program was used for analyzing the fluorescence intensity results of each cell. The results were analyzed by using ImageJ/Imaris software and the mean values were expressed as arbitrary unit.

Axiocam 702 mono is a high-performance sCMOS microscope camera with 2.3 megapixels and a sensor size of 1/1.2″ (diagonal 13.3 mm). By the Zeiss company, the camera was adapted to the laser confocal microscope, and 3D bride field images of JC-1, DHR23, DCFH-DA and MitoROS were taken by high performance camera (Axiocam 702 mono) in CISP + CURC groups.

### Assay of reduced glutathione (RGS) level and glutathione peroxidase (GSPx) activity

RGS and lipid peroxidation (malondialdehyde, MDA) levels of the Hep2 cells were spectrophotometrically (Cary 60 UV-Vis Spectrophotometer, Agilent, İzmir, Turkey) analyzed at 412 nm and 532 nm by using the methods of Sedlak and Lindsay^50^ and Placer *et al*.^51^, respectively. RGS and MDA levels in Hep2 cells were expressed as μmol/g protein.

The spectrophotometric (Cary 60 UV) method of Lawrence and Burk^52^ was used in the cells for measuring glutathione peroxidase (GSPx) activity as described in the previous studies^9,10^. The activity of GSPx is expressed as an international unit (IU) of RGS oxidized/min/g protein. The total protein in the supernatant was spectrophotometrically (Shimadzu UV-1800) assessed using the Bradford reagent at 595 nm.

### Western Blot analyses for TRPM2 channel expression

Standard procedures of Western Blot analysis were used for the expression of TRPM2 channel in Hep2 cells as described in previous studies^9,10^. Briefly, each protein sample (25 μg) of TRPM2 and β-actin was separated using SDS-gel electrophoresis and transferred onto a nitrocellulose membrane (0.45 µm). The membrane was incubated in blocking buffer. The membranes were washed with 1x PBS and incubated with primary antibodies of TRPM2 and β-actin overnight at 4 °C. A day after, blots were washed with 1x PBS and incubated with secondary antibodies. The western blot bands were detected by using ECL Western HRP Substrate (Millipore Luminate Forte, USA), and the imaging was performed by using Gel Imagination System (G:Box, Syngene, UK).

### Statistical analyses

The values have been expressed in mean ± standard deviation (SD). Fisher’s least significant difference (LSD) test was used in the four groups for determining statistical significance (SPSS program, version 18.0, software, SPSS. Chicago, IL, USA). The presence of statistical significance was detected as p < 0.05 by using a Mann-Whitney U test.

### Compliance with ethical standards

This article does not contain any studies with human participants performed by any of the authors.

## Supplementary information

Supplementary Figure 1

## Data Availability

All methods in the manuscript were performed in accordance with the relevant guidelines and regulations of Suleyman Demirel University (Isparta, Turkey) by including a statement in the methods section to this effect. The dataset and analyses were generated in the BSN Health, Analyses, Innovation, Consultancy, Organization, Agriculture, Industry and Trade Limited Company, Göller Bölgesi Teknokenti, Isparta, Turkey and are available from the corresponding authors on reasonable request. Graphics in the manuscript were prepared by the corresponding author (Prof. Dr. Mustafa Nazıroğlu).

## References

[1] Parkin DM, Bray F, Ferlay J, Pisani P (2005). Global cancer statistics, 2002. CA Cancer J Clin.

[2] Coskun H (2019). Prognosis of subglottic carcinoma: Is it really worse?. Head Neck.

[3] Sharma H, Sen S, Singh N (2005). Molecular pathways in the chemosensitization of cisplatin by quercetin in human head and neck cancer. Cancer Biol Ther..

[4] Lv X, Song DM, Niu YH, Wang BS (2016). Inhibition of heme oxygenase-1 enhances the chemosensitivity of laryngeal squamous cell cancer Hep-2 cells to cisplatin. Apoptosis.

[5] Xie Q (2018). TAT-fused IP3R-derived peptide enhances cisplatin sensitivity of ovarian cancer cells by increasing ER Ca^2+^ release. Int J Mol Med..

[6] Nazıroğlu M, Braidy N (2017). Thermo-sensitive TRP channels: Novel targets for treating chemotherapy-induced peripheral pain. Front Physiol..

[7] Yoon CY (2011). The histone deacetylase inhibitor trichostatin A synergistically resensitizes a cisplatin resistant human bladder cancer cell line. J Urol..

[8] Briehl MM, Tome ME, Wilkinson ST, Jaramillo MC, Lee K (2014). Mitochondria and redox homoeostasis as chemotherapeutic targets. Biochem Soc Trans..

[9] Sakallı Çetin E, Nazıroğlu M, Çiğ B, Övey İS, Aslan Koşar P (2017). Selenium potentiates the anticancer effect of cisplatin against oxidative stress and calcium ion signaling-induced intracellular toxicity in MCF-7 breast cancer cells: Involvement of the TRPV1 channel. J Recept Signal Transduct Res..

[10] Nur G, Nazıroğlu M, Deveci HA (2017). Synergic prooxidant, apoptotic and TRPV1 channel activator effects of alpha-lipoic acid and cisplatin in MCF-7 breast cancer cells. J Recept Signal Transduct Res..

[11] Zhu K, Jiang L, Chu Y, Zhang YS (2016). Protective effect of selenium against cisplatin-induced nasopharyngeal cancer in male albino rats. Oncol Lett..

[12] Hardie RC, Minke B (1992). The trp gene is essential for a light-activated Ca^2+^ channel in Drosophila photoreceptors. Neuron.

[13] Kumar VS, Gopalakrishnan A, Nazıroğlu M, Rajanikant GK (2014). Calcium ion–the key player in cerebral ischemia. Curr Med Chem..

[14] Nazıroğlu M (2011). TRPM2 cation channels, oxidative stress and neurological diseases: where are we now?. Neurochem Res..

[15] Nazıroğlu M (2007). New molecular mechanisms on the activation of TRPM2 channels by oxidative stress and ADP-ribose. Neurochem Res..

[16] Hara Y (2002). LTRPC2 Ca2+-permeable channel activated by changes in redox status confers susceptibility to cell death. Mol Cell..

[17] Nazıroğlu M, Lückhoff A (2008). Effects of antioxidants on calcium influx through TRPM2 channels in transfected cells activated by hydrogen peroxide. J Neurol Sci..

[18] Zendehdel E (2019). The molecular mechanisms of curcumin’s inhibitory effects on cancer stem cells. J Cell Biochem..

[19] Schweyer S, Soruri A, Heintze A, Radzun HJ, Fayyazi A (2004). The role of reactive oxygen species in cisplatin-induced apoptosis in human malignant testicular germ cell lines. Int J Oncol..

[20] Seve P, Dumontet C (2005). Chemoresistance in non-small cell lung cancer. Curr Med Chem Anticancer Agents..

[21] Xu X, Chen D, Ye B, Zhong F, Chen G (2015). Curcumin induces the apoptosis of non-small cell lung cancer cells through a calcium signaling pathway. Int J Mol Med..

[22] Joshi DC, Bakowska JC (2011). Determination of mitochondrial membrane potential and reactive oxygen species in live rat cortical neurons. J Vis Exp.

[23] Keil VC, Funke F, Zeug A, Schild D, Müller M (2011). Ratiometric high-resolution imaging of JC-1 fluorescence reveals the subcellular heterogeneity of astrocytic mitochondria. Pflugers Arch..

[24] An X (2019). Increasing the TRPM2 channel expression in Human Neuroblastoma SH-SY5Y cells augments the susceptibility to ROS-induced cell death. Cells.

[25] Baş E, Nazıroğlu M (2019). Selenium attenuates docetaxel-induced apoptosis and mitochondrial oxidative stress in kidney cells. Anti-Cancer Drugs..

[26] Uguz AC (2012). Melatonin potentiates chemotherapy-induced cytotoxicity and apoptosis in rat pancreatic tumor cells. J Pineal Res..

[27] Nazıroglu M (2009). Role of selenium on calcium signaling and oxidative stress-induced molecular pathways in epilepsy. Neurochem Res..

[28] Avery JC, Hoffmann PR (2018). Selenium, selenoproteins, and immunity. Nutrients.

[29] Kheradpezhouh E, Barritt GJ, Rychkov GY (2016). Curcumin inhibits activation of TRPM2 channels in rat hepatocytes. Redox Biol..

[30] Epstein J, Sanderson IR, Macdonald TT (2010). Curcumin as a therapeutic agent: the evidence from *in vitro*, animal and human studies. Br J Nutr..

[31] Belrose JC, Xie YF, Gierszewski LJ, MacDonald JF, Jackson MF (2012). Loss of glutathione homeostasis associated with neuronal senescence facilitates TRPM2 channel activation in cultured hippocampal pyramidal neurons. Mol Brain.

[32] Özgül C, Nazıroğlu M (2012). TRPM2 channel protective properties of N-acetylcysteine on cytosolic glutathione depletion dependent oxidative stress and Ca^2+^ influx in rat dorsal root ganglion. Physiol Behav..

[33] Öz A, Çelik Ö (2016). Curcumin inhibits oxidative stress-induced TRPM2 channel activation, calcium ion entry and apoptosis values in SH-SY5Y neuroblastoma cells: Involvement of transfection procedure. Mol Membr Biol..

[34] Nazıroğlu M (2019). Albumin evokes Ca^2+^-induced cell oxidative stress and apoptosis through TRPM2 channel in renal collecting duct cells reduced by curcumin. Sci Rep.

[35] Uğuz AC, Öz A, Nazıroğlu M (2016). Curcumin inhibits apoptosis by regulating intracellular calcium release, reactive oxygen species and mitochondrial depolarization levels in SH-SY5Y neuronal cells. J Recept Signal Transduct Res..

[36] Bardak H, Uğuz AC, Bardak Y (2017). Curcumin regulates intracellular calcium release and inhibits oxidative stress parameters, VEGF, and caspase-3/-9 levels in human retinal pigment epithelium cells. Physiol Int..

[37] Baliga MS (2012). Curcumin, an active component of turmeric in the prevention and treatment of ulcerative colitis: preclinical and clinical observations. Food Funct..

[38] Öz A, Çelik Ö, Övey İS (2017). Effects of different doses of curcumin on apoptosis, mitochondrial oxidative stress and calcium influx in DBTRG glioblastoma cells. J Cell Neurosci Oxid Stress.

[39] Uğuz AC (2009). Selenium modulates oxidative stress induced cell apoptosis in human myeloid HL-60 cells via regulation of caspase-3, -9 and calcium influx. J Membr Biol.

[40] Peng F, Zhang H, Du Y, Tan P (2018). Cetuximab enhances cisplatin-induced endoplasmic reticulum stress-associated apoptosis in laryngeal squamous cell carcinoma cells by inhibiting expression of TXNDC5. Mol Med Rep..

[41] Mortezaee K (2019). Mechanisms of apoptosis modulation by curcumin: Implications for cancer therapy. J Cell Physiol..

[42] Su CC (2006). Curcumin-induced apoptosis of human colon cancer colo 205 cells through the production of ROS, Ca^2+^ and the activation of caspase-3. Anticancer Res..

[43] Cabelguenne A (2001). Glutathione-associated enzymes in head and neck squamous cell carcinoma and response to cisplatin-based neoadjuvant chemotherapy. Int J Cancer..

[44] Pereira DL, Dos Santos Ferreira AC, de Faria GP, Kwee JK (2015). Autophagy interplays with apoptosis and cell cycle regulation in the growth inhibiting effect of Trisenox in HEP-2, a laryngeal squamous cancer. Pathol Oncol Res..

[45] Polzin D (2010). Decreased renal corin expression contributes to sodium retention in proteinuric kidney diseases. Kidney Int..

[46] Hu A (2014). Curcumin suppresses invasiveness and vasculogenic mimicry of squamous cell carcinoma of the larynx through the inhibition of JAK-2/STAT-3 signaling pathway. Am J Cancer Res..

[47] McHugh D, Flemming R, Xu SZ, Perraud AL, Beech DJ (2003). Critical intracellular Ca2+ dependence of transient receptor potential melastatin 2 (TRPM2) cation channel activation. J Biol Chem..

[48] Kraft R, Grimm C, Frenzel H, Harteneck C (2006). Inhibition of TRPM2 cation channels by N-(p-amylcinnamoyl)anthranilic acid. Br J Pharmacol..

[49] Togashi K, Inada H, Tominaga M (2008). Inhibition of the transient receptor potential cation channel TRPM2 by 2-aminoethoxydiphenyl borate (2-APB). Br J Pharmacol..

[50] Sedlak J, Lindsay RHC (1968). Estimation of total, protein bound and non-protein sulfhydryl groups in tissue with Ellmann’ s reagent. Anal Biochem..

[51] Placer ZA, Cushman L, Johnson BC (1966). Estimation of products of lipid peroxidation (malonyl dialdehyde) in biological fluids. Anal Biochem.

[52] Lawrence RA, Burk RF (2012). Glutathione peroxidase activity in selenium-deficient rat liver. Biochem Biophys Res Commun..

