# Supplementary material for: Curcumin enhances cisplatin-induced human laryngeal squamous cancer cell death through activation of TRPM2 channel and mitochondrial oxidative stress
Source: Sci Rep. 2019 Nov 28;9:17784. doi: 10.1038/s41598-019-54284-x (PMC6882809; doi:10.1038/s41598-019-54284-x)

# **Curcumin enhances cisplatin-induced human laryngeal squamous cancer cell death through activation of TRPM2 channel and mitochondrial oxidative stress**

**Sinem Gökçe Kütük^1^, Gökçen Gökçe^2^, Mustafa Kütük^3^, Hacer Esra Gürses Cila^4^, Mustafa Nazıroğlu^5,6^**

**Supplementary Figure 1**. Full length Western blots of TRPM2 channel and β-actin for Figure 1 (n=3).


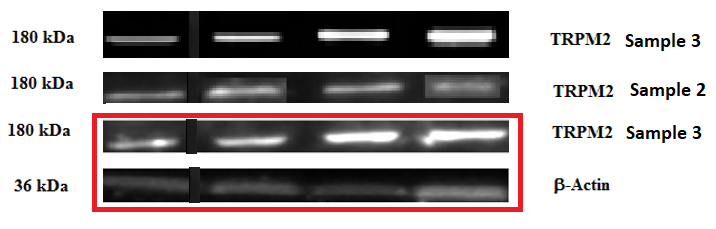

Supplement: Supplementary file 1 — Supplementary Figure 1 [file 41598_2019_54284_MOESM1_ESM.docx]
